# Drought‐induced changes in flow regimes lead to long‐term losses in mussel‐provided ecosystem services

**DOI:** 10.1002/ece3.2366

**Published:** 2016-08-14

**Authors:** Caryn C. Vaughn, Carla L. Atkinson, Jason P. Julian

*Ecology and Evolution* 2015; 5(6): 1291–1305

doi: 10.1002/ece3.1442

**Table 1:** Mussel biomass and excretion rates used to estimate nitrogen and phosphorus recycling rates and storage

Species	Mean soft tissue dry mass (g/m^2^)						Ammonium excretion (*μ*g L /h/g)			Phosphate excretion (*μ*g L /h/g)			Clearance rate (L/h/g)		
	Upstream sites			Downstream sites											
	1991	2003	2011	1991	2003	2011	15**°**	25**°**	35**°**	15**°**	25**°**	35**°**	15**°**	25**°**	35**°**
*Actinonaias ligamentina*	21.00	6.47	0.00	55.08	23.45	38.19	0.479	1.019	1.636	0.261	0.269	0.410	0.452	3.210	2.363
*Amblema plicata*	21.31	5.21	11.68	31.35	12.80	10.31	0.248	0.556	0.766	0.206	0.229	0.211	0.612	1.211	3.572
*Ellipsaria lineolata*	0.00	0.00	0.00	2.62	11.19	2.61	0.366	0.909	1.362	0.239	0.298	0.359	1.000	3.600	6.600
*Fusconaia flava*	3.05	0.78	0.26	3.65	1.51	0.21	0.230	0.829	0.913	0.204	0.326	0.229	0.709	3.423	7.475
*Lampsilis cardium*	7.70	5.89	1.61	4.42	7.49	1.62	0.578	0.988	2.389	0.299	0.539	0.534	1.810	4.707	2.343
*Lampsilis teres*	0.41	1.83	1.09	0.00	0.00	0.00	0.366	0.909	1.362	0.239	0.298	0.359	1.000	3.600	6.600
*Obliquaria reflexa*	0.25	0.00	0.00	0.76	0.00	0.38	0.216	1.041	1.376	0.208	0.200	0.220	0.694	4.010	9.587
*Potamilus purpuratus*	0.58	0.00	0.00	0.93	13.58	2.11	0.366	0.909	1.362	0.239	0.298	0.359	1.000	3.600	6.600
*Ptychobranchus occidentalis*	0.25	2.38	0.54	0.32	0.00	0.00	0.366	0.909	1.362	0.239	0.298	0.359	1.000	3.600	6.600
*Quadrula pustulosa*	5.49	0.75	0.00	9.13	2.38	0.63	0.167	0.757	1.362	0.186	0.305	0.534	2.224	4.989	15.451
*Tritogonia verrucosa*	3.94	1.04	0.41	1.64	0.00	0.74	0.784	0.936	1.572	0.239	0.298	0.359	1.000	3.600	6.600
*Truncilla truncata*	0.21	0.00	0.00	0.43	1.23	0.19	0.340	1.041	1.077	0.294	0.311	0.389	0.911	7.603	7.614

